# DFT analysis of structural, electronic and optical properties of Ni and Zn doped CoS counter electrode for dye sensitized solar cells

**DOI:** 10.1038/s41598-025-19663-7

**Published:** 2025-10-10

**Authors:** Dereje Gelanu Dadi, Megersa Wodajo Shura, Fekadu Gochole

**Affiliations:** 1 Department of Applied Physics, Adama Science and Technology University, PO.Box. 1888, Adama, Ethiopia; 2 Department of Material Science and Engineering, Adama Science and Technology University, PO.Box. 1888, Adama, Ethiopia

**Keywords:** Density functional theory, Cobalt sulfide, Codope, Electronic Properties, Optical properties, Counter electrode, Dye sensitized solar cells, Materials science, Condensed-matter physics, Theory and computation

## Abstract

In this study, first principles calculations are employed to investigate the structural, electronic, and optical properties of $\hbox {(Ni, Zn)}_x\hbox {Co}_{1-x}$S in its tetragonal phase. The optimized lattice parameters and negative defect formation energies confirm structural integrity and thermodynamic stability across doping levels. The calculations are performed using both the GGA and the HSE06 hybrid functional, ensuring an accurate description of electronic band structure. The system retains a direct band gap in all doped configurations, with a systematic reduction upon doping most pronounced in the co-doped case, demonstrating effective band gap engineering. Ni doping enhances electron localization through stronger Ni–S bonding, while Zn doping promotes electron delocalization, collectively improving charge transport. The band edges are dominated by hybridized Co(3d), Ni(3d), and S(3p) states, with Zn(4s) modulating valence band characteristics. A notable reduction in the effective masses of electrons and holes upon co-doping indicates enhanced carrier mobility and improved conductivity. Optical calculations reveal increased dielectric constant, strong absorption in the UV–visible range, and enhanced electrical conductivity, particularly in co-doped systems. These results establish $\hbox {(Ni, Zn)}_x\hbox {Co}_{1-x}$S as a highly tunable, stable, and efficient material, positioning it as a promising low-cost alternative to platinum-based counter electrodes in dye sensitized solar cells. Moreover, this work offers crucial guidance for experimentalists in tuning CoS properties via doping for efficient dye-sensitized solar cells.

## Introduction

The energy demand worldwide has increased due to population growth, industrialization, and technological progress. Despite this developing demand, fossil fuels constitute the dominant energy source, contributing significantly to greenhouse gas emissions and environmental degradation^1,2^. This situation underscores the urgent need for sustainable and clean energy solutions as nations strive to combat climate change and transition to greener economies. The future of energy depends more on renewable sources such as solar, wind, and hydropower, which can help meet rising energy needs while minimizing the carbon footprint^3,4^.

Among photovoltaic technologies, dye-sensitized solar cells (DSSCs) have attracted considerable attention due to their low production cost, simple fabrication process, and relatively high efficiency under diffuse light conditions^5^. A typical DSSC consists of five key components: a transparent conducting oxide (TCO) coated substrate, a mesoporous wide-bandgap semiconductor (commonly $\hbox {TiO}_{2}$), a photosensitive dye, an electrolyte containing a redox mediator (typically $\hbox {I}^{-}$/$\hbox {I}_{3}^{-}$), and a counter electrode (CE) responsible for catalyzing the reduction of the electrolyte and regenerating the oxidized dye^6,7^. The performance of the CE significantly influences the overall efficiency of the device, and platinum (Pt) has traditionally been used due to its excellent electrical conductivity and electrocatalytic activity. However, the high cost, scarcity, and long-term instability of Pt in iodide-based electrolytes have motivated extensive research into alternative, earth-abundant materials for CE applications^8,9^.

Transition metal chalcogenides (TMCs), particularly transition metal sulfides, have emerged as promising candidates for replacing Pt in DSSCs owing to their high natural abundance, tunable electronic structures, and strong electrocatalytic properties^10,11^. Among these, cobalt sulfide (CoS) has drawn significant interest due to its favorable physical and chemical characteristics, including good electrical conductivity, excellent catalytic activity toward the redox couple, and environmental compatibility^12,13^. Cobalt sulfide is a member of the transition metal chalcogenide family, existing in multiple phases such as CoS, $\hbox {Co}_{2}$S, $\hbox {Co}_{3}\hbox {S}_{4}$, and $\hbox {Co}_{9}\hbox {S}_{8}$^14,15^. Its phase chemistry is complex due to non-stoichiometry, variable oxidation states, and the coexistence of different structural and compositional variants^14,16,17^. Experimental studies have demonstrated that pristine and doped CoS nanostructures and composites exhibit comparable or even superior catalytic performance to Pt in DSSCs^13,18,19^.

Despite growing experimental interest, theoretical investigations into the fundamental properties of transitional metal-doped CoS remain limited. Conventional density functional theory (DFT) methods, like the generalized gradient approximation (GGA), often struggle to accurately capture the electronic structure of transition metal monosulfides. While hybrid functionals and GW approximations provide greater accuracy, they incur a substantially higher computational cost^20–22^. Consequently, there is a pressing need for comprehensive first-principles studies to reliably assess the structural, electronic, and optical properties of CoS-based systems. Recently, Dadi et al. investigated CoS as a CE material for DSSCs using DFT, highlighting its promising electrocatalytic activity and stability^23^. To further enhance the performance of CoS while reducing material costs, transition metal doping strategies, particularly with nickel (Ni) and zinc (Zn), have been explored. Single doping with Ni or Zn, as well as co-doping with both elements, can effectively tune the electronic structure, improve electrical conductivity, and optimize surface catalytic sites in CoS, thereby boosting its efficiency as a CE in DSSCs. However, the fundamental mechanisms by which these dopants influence the electronic and optical behavior of CoS remain insufficiently understood from a theoretical perspective.

In this study, we employ first-principles DFT calculations to systematically investigate the structural, electronic, and optical properties of $\hbox {(Ni, Zn)}_{x}\hbox {Co}_{1-x}$S alloys. By analyzing the effects of both single and co-doping on the band structure, density of states, and optical response, we aim to elucidate the underlying physical mechanisms and evaluate the potential of doped CoS as a high-performance, low-cost alternative to conventional CE materials in DSSCs.

## Computational methodology

In this study, first-principles calculations using the Quantum ESPRESSO code^24,25^ were employed to investigate the crystal structure, electronic and optical properties, and electron cloud density of Ni, Zn, and co-doped CoS materials. The electron wave function was expanded in a plane-wave basis set, employing the pseudopotential method to approximate core electrons. The exchange-correlation energy was treated using the PBEsol functional (Perdew-Burke-Ernzerhof) under the generalized gradient approximation (GGA)^26^, and the Heyd-Scuseria-Ernzerhof (HSE)^27^ hybrid functional for improved electronic structure description. Conventional DFT calculations on a supercell (SC) with random alloy configurations can lead to folded and fragmented band structures that obscure the true electronic dispersion. To address this challenge, our study employs the SC approach combined with band unfolding techniques to obtain more physically meaningful band structures, effectively mapping the electronic states onto the primitive host lattice. This allows us to extract unfolded band dispersions that better represent the quasi-particle excitations and provide clearer insight into the evolution of band edges of alloys^22,28^. Kinetic energy cut-offs of 70 Ry for wave functions and 560 Ry for charge density were selected based on convergence tests to ensure accurate evaluations of the wave functions and precise representation of the electronic density. The structural models were initialized using crystallographic information from the Materials Project database. A $2 \times 2 \times 1$ supercell was generated using Phonopy to enable substitutional doping while preserving primitive lattice symmetry. Geometry optimization was carried out using the Broyden-Fletcher-Goldfarb-Shanno (BFGS) minimization method to achieve the most stable configuration of the $\hbox {(Ni, Zn)}_{x}\hbox {Co}_{1-x}$S system, where *x* represented values of 0.125, 0.25, and 0.375 compositions. Brillouin zone integration was performed using a Monkhorst-Pack k-point grid of $9 \times 9 \times 7$ with ultrasoft pseudopotentials for structural and energetic calculations. It is essential to note that spin-orbit coupling (SOC) was excluded from the calculations to prevent any overestimation of energy values in the generated properties^29^. Electronic band structures were calculated using a dense, high-symmetry k-point mesh (generated by XcySDen), while the density of states was determined using a finer 22 $\times$ 22 $\times$ 20 k-point sampling set. The optical characteristics were evaluated using norm-conserving pseudopotentials, which provide an accurate description of core-valence interactions, ensuring reliable modeling of electronic and optical responses in the system. The valence electron configurations of Co, Ni, Zn, and S were considered to be 3$\hbox {d}^{7}$4$\hbox {s}^{2}$, 3$\hbox {d}^{8}$4$\hbox {s}^{2}$, 3$\hbox {d}^{10}$4$\hbox {s}^{2}$, and 3$\hbox {s}^{2}$3$\hbox {p}^{4}$, respectively. This comprehensive computational approach enabled a detailed analysis of the electronic and optical properties of the Ni, Zn, and co-doped CoS systems.

## Results and discussion

### Structural properties and stability

**Fig. 1:**
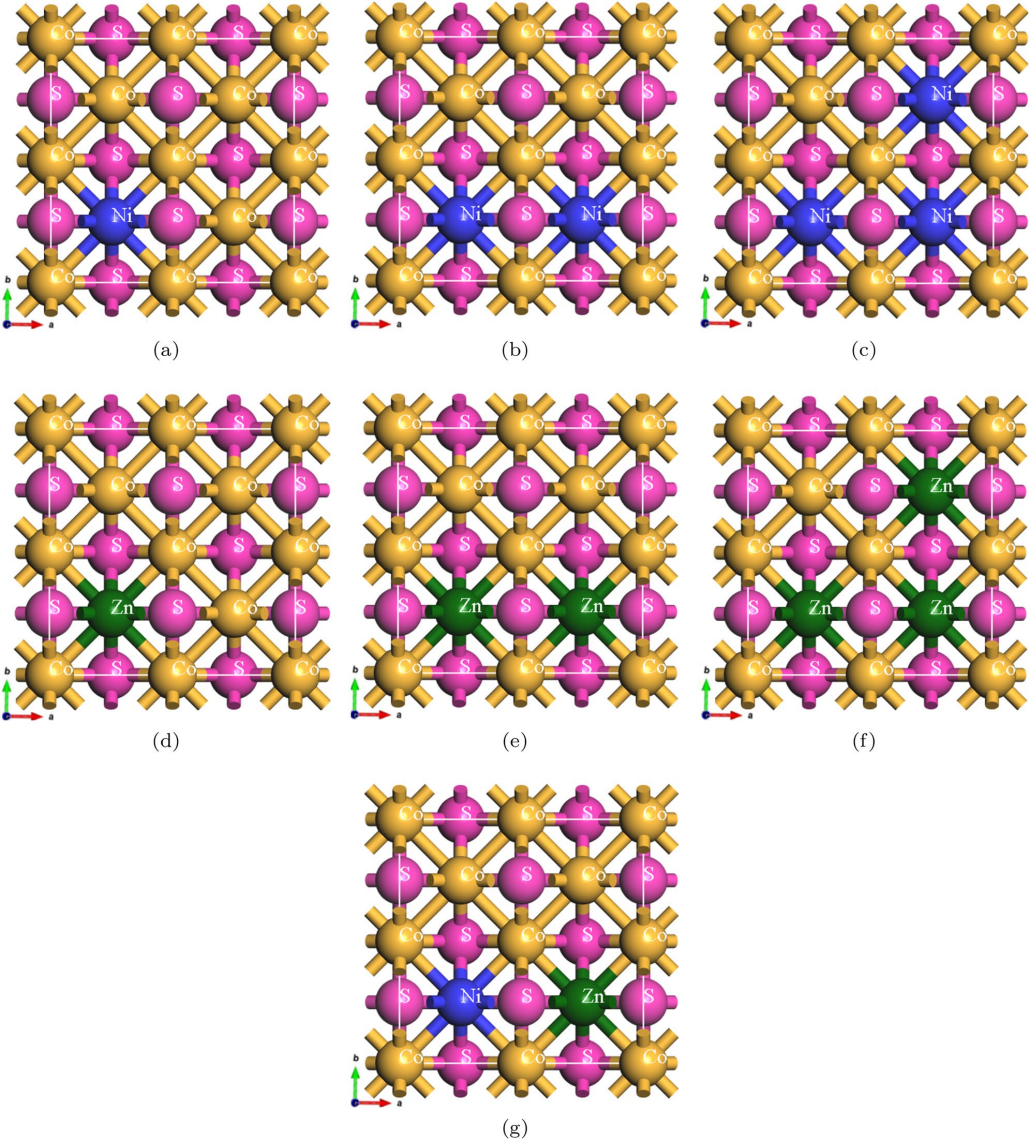
Top view of 2 $\times$ 2 $\times$ 1 supercell crystal structure of: (**a**) $\hbox {Ni}_{0.125}\hbox {Co}_{0.875}$S, (**b**) $\hbox {Ni}_{0.250}\hbox {Co}_{0.750}$S, (**c**) $\hbox {Ni}_{0.375}\hbox {Co}_{0.625}$S, (**d**) $\hbox {Zn}_{0.125}\hbox {Co}_{0.875}$S, (**e**) $\hbox {Zn}_{0.375}\hbox {Co}_{0.625}$S, (**f**) $\hbox {Zn}_{0.250}\hbox {Co}_{0.750}$S, (**g**) $\hbox {(Ni, Zn)}_{x}\hbox {Co}_{1-x}$S (*x* = 0.25). Structures were visualized using BIOVIA Materials Studio v20.1.0.2728^31^.https://www.3ds.com/products-services/biovia/products/molecular-modeling-simulation/biovia-materials-studio/.

Figure 1 illustrates the crystal structures of $\hbox {Ni}_x\hbox {Co}_{1-x}$S, $\hbox {Zn}_x\hbox {Co}_{1-x}$S, and $\hbox {(Ni, Zn)}_x\hbox {Co}_{1-x}$S (with *x* values of 0.125, 0.25, 0.375, and 0.25 for single and co-doped samples, respectively). The $\hbox {(Ni, Zn)}_{x}\hbox {Co}_{1-x}$S compound exhibits a tetragonal structure with a $\hbox {P}_4$/nmm space group, which imparts unique physical properties due to its high symmetry. This structure substantially influences its electronic, optical, and electron cloud density properties. The substitution of cations modifies the local atomic arrangement, impacting the band structure and charge distribution, thus enabling precise adjustment of the material’s characteristics for use in optoelectronic devices, photocatalytic processes, and advanced solar cell technologies^30^. The lengths of the bonds in $\hbox {(Ni, Zn)}_{x}\hbox {Co}_{1-x}$S critically affect its electronic and optical properties by modifying the atomic interactions. The ionic radii and electronic configurations of the dopants play a key role in changing the bonding environment^32^. The complex electronic configuration of nickel, characterized by partially filled d orbitals, introduces additional energy levels within the band gap, improving charge distribution and interaction with specific energy photons. In contrast, zinc’s simpler configuration and larger ionic radius create fewer impurity states, promoting charge delocalization and improved carrier mobility. Co-doping balances the effects of Ni and Zn, optimizing the electronic structure. Nickel fine-tunes the band edges and introduces new states, while zinc facilitates charge transfer and reduces localization, enhancing charge mobility and electronic performance. Overall, shorter bond lengths typically lead to stronger covalent interactions, narrowing the band gap and increasing the conductivity, while longer bond lengths may result in localized states that impede electron mobility. Furthermore, variations in bond length influence optical properties, affecting the absorption spectrum and the wavelengths of light that can be absorbed, which is critical for photovoltaics and optoelectronics^33^. Adjustments in bond lengths also alter dielectric properties, affecting polarization in response to electric fields. Understanding these interactions is essential for tailoring $\hbox {(Ni, Zn)}_{x}\hbox {Co}_{1-x}$S for specific electronic and optical applications, making it a promising candidate for advanced technology. The lattice parameters and bond lengths were calculated based on the optimized single Ni, Zn, and co-doped CoS alloys and agree with the reported in recent studies^15,34^. The calculated lattice parameters and the bond length results are presented in Table 1. The optimized $\hbox {(Ni, Zn)}_{x}\hbox {Co}_{1-x}$S crystal structures, as obtained by calculation from the Quantum ESPRESSO package, were inspected by first converting the Output files into.xsf file format with the assistance of XCrySDen. The files were then imported into BIOVIA Materials Studio for the purpose of generating and displaying the 3D crystal structures graphically^31^.

**Table 1:** Summary of calculated lattice constants and bond lengths for $\hbox {(Ni, Zn)}_{x}\hbox {Co}_{1-x}$S compounds.

Material	Lattice Constants (Å)		Bond Lengths (Å)				
	$a = b$	*c*	Co–S	Ni–S	Zn–S	Ni–Ni	Zn–Zn
CoS	3.53	4.8^15,23^	2.15	–	–	–	–
$\hbox {Ni}_{0.125}\hbox {Co}_{0.875}$S	3.54	4.80	2.13	2.19	–	–	–
$\hbox {Ni}_{0.250}\hbox {Co}_{0.750}$S	3.56	4.70	2.17	2.19	–	3.56	–
$\hbox {Ni}_{0.375}\hbox {Co}_{0.625}$S	3.58	4.68	2.18	2.17	–	3.58	–
$\hbox {Zn}_{0.125}\hbox {Co}_{0.875}$S	3.56	4.82	2.30	–	2.14	–	–
$\hbox {Zn}_{0.250}\hbox {Co}_{0.750}$S	3.61	4.79	2.32	–	2.27	–	3.67
$\hbox {Zn}_{0.375}\hbox {Co}_{0.625}$S	3.67	4.83	2.32	–	2.27	–	3.61
$\hbox {(Ni, Zn)}_{0.25}\hbox {Co}_{0.75}$S	3.59	4.76	2.24	2.18	2.27	–	–

Moreover, the thermodynamic stability of $\hbox {(Ni, Zn)}_{x}\hbox {Co}_{1-x}$ alloys was investigated through the calculation of defect formation energies. The formation energy is a key metric in alloy thermodynamics, determining the stability of a compound relative to its constituent elements in their standard states. The defect formation energy ($E_f$) for the doped systems is expressed as^35^.

1 $$\begin{aligned} E_f = E_{M:\text {CoS}} - E_{\text {CoS}} + n_i(\mu _{\text {Co}} - \mu _{M}), \end{aligned}$$

where $E_{M:\text {CoS}}$ is the total energy of the dopant atoms *M* (Ni, Zn, or co-dopants) substitute Co sites, $E_{\text {CoS}}$ is the energy of pristine, $n_i$ is the number of dopant atoms, and $\mu _{\text {Co}}$ and $\mu _{M}$ are the chemical potentials of cobalt and the dopant element (which represents the energy level of the reservoirs from which atoms are exchanged^36^, respectively. This formulation accounts for the energetic cost of replacing Co atoms with dopants under S-rich conditions, providing a measure of thermodynamic stability. A more negative $E_f$ indicates a more stable doped system. The calculated defect formation energies for Ni, Zn, and co-doped CoS at various concentrations are presented in Table 2. As the concentration of either Ni or Zn increases, the total energy of the system becomes more negative, indicating improved thermodynamic stability. In Ni-doped CoS, the defect formation energy decreases with higher concentrations, accompanied by increasingly negative formation energies per atom. Similarly, Zn-doped CoS exhibits a comparable trend, where the defect formation energies decrease with increasing dopant concentration. Notably, Ni doping results in more negative formation energies compared to Zn doping at similar concentrations, suggesting that Ni is more effective in stabilizing the CoS lattice.

**Table 2:** Defect formation energies for Ni, Zn, and co-doped CoS systems.

Systems	Total Energy (Ry)	$\hbox {E}_{f}$ (Ry)	$\hbox {E}_{f}$ (Ry/atom)
$\hbox {Ni}_{0.125}\hbox {Co}_{0.875}$	$-$2369.8002	$-$0.9776	$-$0.0611
$\hbox {Ni}_{0.250}\hbox {Co}_{0.750}$S	$-$2370.7886	$-$1.9552	$-$0.1222
$\hbox {Ni}_{0.375}\hbox {Co}_{0.625}$S	$-$2371.7685	$-$2.9240	$-$0.1828
$\hbox {Zn}_{0.125}\hbox {Co}_{0.875}$S	$-$2366.8001	$-$0.9519	$-$0.0600
$\hbox {Zn}_{0.250}\hbox {Co}_{0.750}$S	$-$2367.7886	$-$1.9037	$-$0.1190
$\hbox {Zn}_{0.375}\hbox {Co}_{0.625}$	$-$2368.7780	$-$2.8564	$-$0.1785
$\hbox {(Ni, Zn)}_{0.25}\hbox {Co}_{0.75}$S	$-$2368.5600	$-$2.6972	$-$0.1686

The co-doped systems exhibit the most negative defect formation energy, highlighting that co-doping significantly enhances stability compared to single-dopant scenarios. These findings show that while higher dopant concentrations may lead to elevated formation energies due to lattice strain and atomic repulsion, the system still maintains thermodynamic stability, particularly at optimal concentrations that enhance material performance.

### Electronic properties

The effective band structure method is employed to compute the unfolded band structures^22^ of $\hbox {(Ni, Zn)}_{x}\hbox {Co}_{1-x}$S alloys, effectively removing the influence of long-range disorder and recovering the coherent band dispersion in the primitive Brillouin zone. The degree of preservation or degradation of band features as a function of composition, band index, and k-point^28^ is revealed by the resulting effective band structure, and a rapid breakdown of valence band Bloch character in $\hbox {(Ni, Zn)}_x\hbox {Co}_{1-x}$S is observed. Investigating the effects of Ni, Zn, and codoping on the band structure is crucial to enhancing their potential in electronic technology applications. Hybrid functional methods such as GGA+U, HSE_x_, and GW^20,21,37^, which incorporate both local DFT exchange-correlation and a fraction of exact Hartree–Fock (HF) exchange, tend to overestimate the band gaps of materials relative to experimental values. The calculated band structure of $\hbox {(Ni, Zn)}_{x}\hbox {Co}_{1-x}$S, analyzed along the high-symmetry paths within the Brillouin zone using GGA and the HSE06 hybrid functional, shows that both the conduction band minimum (CBM) and the valence band maximum (VBM) are situated at the $\Gamma$-point, indicating a direct bandgap.

**Fig. 2:**
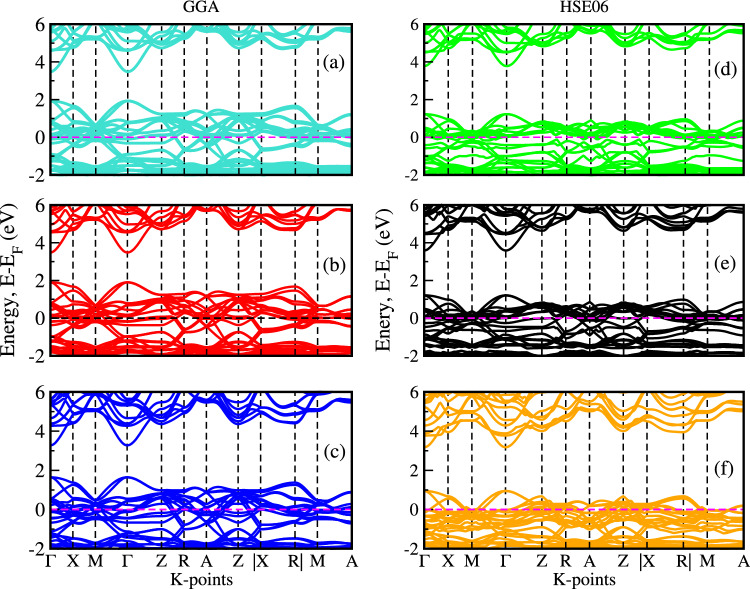
Electronic band structures of $\hbox {Ni}_{0.125}\hbox {Co}_{0.875}$S, $\hbox {Ni}_{0.25}\hbox {Co}_{0.75}$S, and $\hbox {Ni}_{0.375}\hbox {Co}_{0.625}$S calculated using the GGA functional (**a**–**c**) and the HSE06 hybrid functional (**d**–**f**), respectively.

**Fig. 3:**
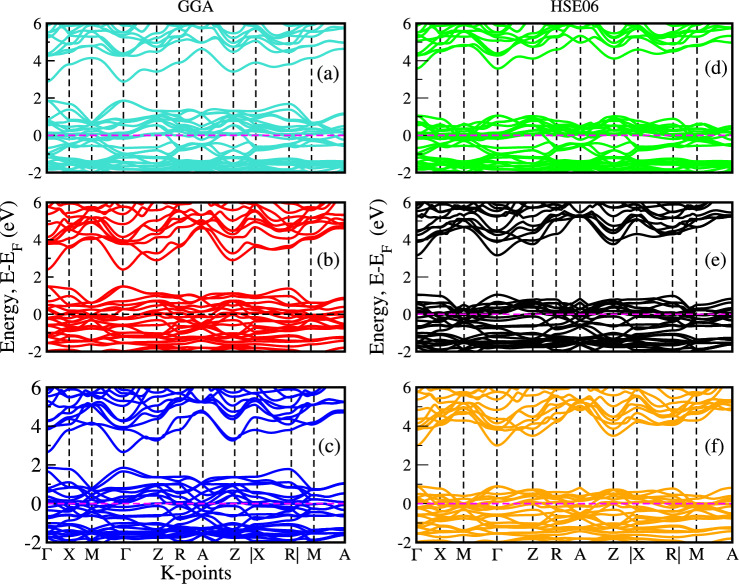
Electronic band structures of $\hbox {Zn}_{0.125}\hbox {Co}_{0.875}$S, $\hbox {Zn}_{0.25}\hbox {Co}_{0.75}$S, and $\hbox {Zn}_{0.375}\hbox {Co}_{0.625}$S calculated using the GGA functional (**a**–**c**) and the HSE06 hybrid functional (**d**–**f**), respectively.

**Fig. 4:**
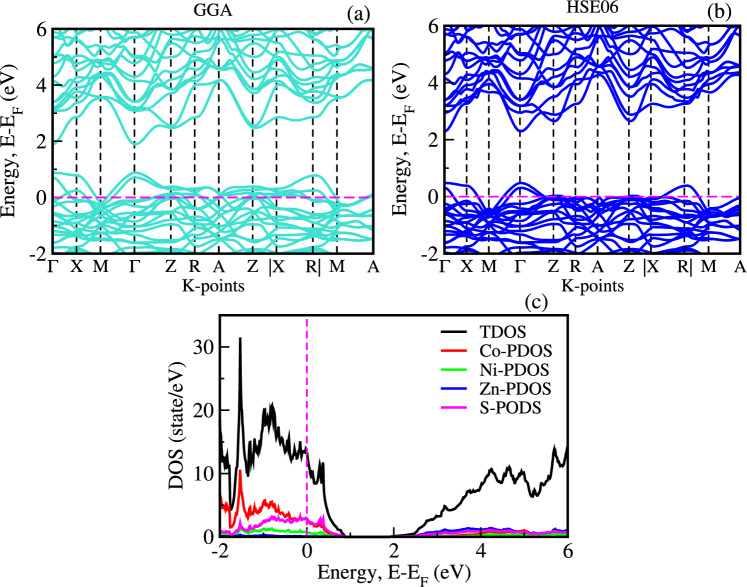
Electronic band structures and density of states (DOS) of $\hbox {(Ni, Zn)}_{x}\hbox {Co}_{1-x}$S ($x = 0.25$).

**Fig. 5:**
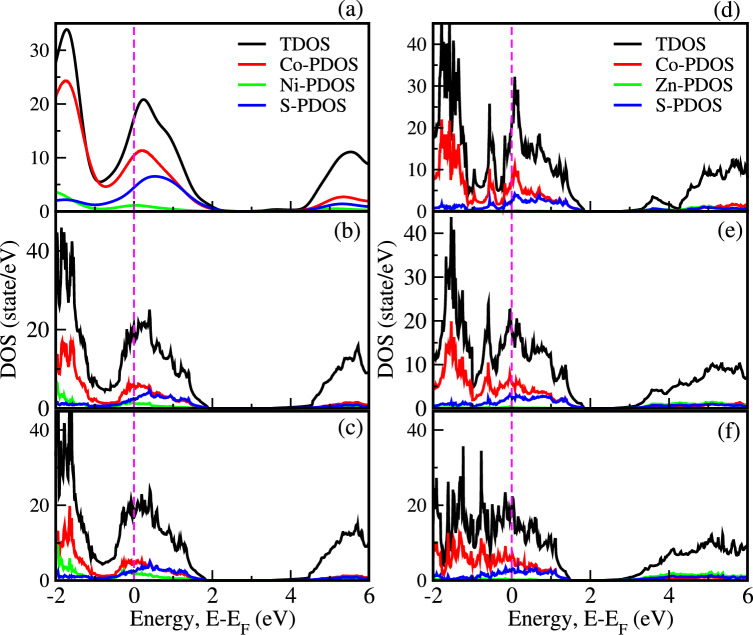
Density of states (DOS) of $\hbox {(Ni, Zn)}_{x}\hbox {Co}_{1-x}$S (x = 0.125, 0.25, and 0.375).

The introduction of Ni and Zn dopants introduces impurity levels within the valence and conduction bands, leading to substantial modifications in the characteristics of the band gap^38^. Figures 2,3,4 show the calculated unfolded band structures of Ni, Zn, and co-doped CoS using both the GGA and HSE06 functionals. As shown in Fig. 2 (a - c), for the GGA functional, the band gap of Ni-doped CoS decreases from 1.628 eV to 1.550 eV with doping concentrations of 0.125, 0.25, and 0.375, respectively. A similar trend is observed when using the HSE06 hybrid functional, which provides a more accurate description of the electronic structure compared to GGA. As shown in Fig. 2 (d–f), for the HSE06 functional, the band gap of Ni-doped CoS decreases from 1.84 eV to 1.75 eV with increasing the same doping concentrations. This gradual reduction indicates the influence of Ni doping on the electronic structure, consistent with the GGA results but with slightly larger absolute band gap values, as typically expected with hybrid functionals. In Ni-doped CoS, the reduction is primarily due to the complex d orbitals of Ni, which introduce additional energy levels within the band gap. These intermediate states enhance electron transitions and strengthen interactions with the host crystal’s electronic environment, efficiently lowering the band gap and improving the material’s light absorption capabilities.

Similarly, as shown in Fig. 3(a–f), GGA calculations reveal that Zn doping reduces the band gap of CoS from 1.45 eV to 1.34 eV with increasing Zn concentration, while HSE06 calculations yield a corresponding reduction from 1.68 eV to 1.60 eV and agree with previous reported^15^. In contrast, Zn doping has a more substantial effect on the band gap. The simple electronic configuration of Zn introduces fewer impurity states comparable to Ni, promoting enhanced charge transfer and minimizing lattice strain. It indicates that Zn facilitates a more efficient charge carrier transport mechanism, increasing overall conductivity while narrowing the band gap without introducing as many complex energy levels^39^. Moreover, as shown in Fig. 4(a–b), the electronic bandgap of the co-doped system was computed using the GGA and HSE06 functionals, yielding values of 1.214 eV and 1.61 eV, respectively. The hybrid functional method, which incorporates both local DFT exchange-correlation and a fraction of exact Hartree–Fock (HF) exchange, tends to overestimate the bandgap in $\hbox {(Ni, Zn)}_{x}\hbox {Co}_{1-x}$ systems compared to experimental values and standard GGA calculations.

The analysis of the projected density of states (PDOS) offers insights into the distribution of electronic states across various atomic orbitals. Figure 4(c) indicates that the S(3p) and Zn(4s) orbitals contribute significantly to the conduction band edge due to the empty d-states of $\hbox {Zn}^{2+}$, which enhance hybridization with S(3p). This interaction introduces localized states near the conduction band, promoting charge carrier excitation and improving electrical conductivity^40^. The valence band edge of $\hbox {Ni}_{x}\hbox {Co}_{1-x}$S is primarily composed of hybridized contributions from Co(3d), S(3p), and Ni(3d) orbitals, while the conduction band is dominated by Co(3d) and S(3p) states, with minimal involvement of Ni orbitals, as illustrated in Fig. 5(a–c). Figure 5(d–f) illustrates a similar behavior in $\hbox {Zn}_{x}\hbox {Co}_{1-x}$S, where the conduction band edge is primarily comprised of Co(3d) and S(3p) states, while minimal contributions from Zn(4s) orbitals are noted, despite their significant participation near the conduction edge. As the concentrations of Ni and Zn increase, the impurity levels become more prominent, further modifying the electronic states and causing shifts in energy levels. This ability to finely tune the electronic and optical properties through strategic doping highlights the potential of CoS for use in various applications, particularly in optoelectronics, photovoltaics, and photocatalytic technologies. The precise modulation of cation substitution is essential for optimizing the performance of $\hbox {(Ni, Zn)}_{x}\hbox {Co}_{1-x}$S based materials, paving the way for advanced energy solutions and innovative CE for DSSCs.

The effective mass of charge carriers is a critical parameter that quantifies how electrons and holes respond to applied electric fields within a crystalline material, taking into account the periodic potential of the lattice structure^41^. This parameter plays a vital role in determining the transport properties of semiconducting materials, directly influencing their electrical conductivity, optical absorption characteristics, and overall performance in electronic and optoelectronic applications. The effective masses of charge carriers for Ni, Zn, and co-doped CoS systems were calculated using both the GGA and HSE06 hybrid functional, and the results are summarized in Table 3. The analysis reveals p-type conductivity in all systems, as hole effective masses consistently exceed those of electrons across all doping configurations. Effective masses were derived from the curvature of the energy-wave vector (E-k) dispersion relations near the band edges, using the equation:

2 $$\begin{aligned} m^{*} = \hbar ^{2}\left( \frac{d^{2}E}{dk^{2}}\right) ^{-1} \end{aligned}$$

where $\hbar = 1.05 \times 10^{-34}$ J$\cdot$s is the reduced Planck constant, $E$ is the energy, and $k$ is the wave vector. This relationship allows for the determination of both electron and hole effective masses. The HSE06 calculations demonstrate higher effective masses than GGA across all compositions, correlating with a better description of the band gap and curvature. This systematic increase underscores the critical role of hybrid functionals in predicting carrier dynamics and highlights the tunability of electronic properties for optoelectronic applications.

**Table 3:** Electronic band gaps and effective masses of holes ($m_h^*/m_0$) and electrons ($m_e^*/m_0$) for $\hbox {(Ni, Zn)}_{x}\hbox {Co}_{1-x}$S doped CoS systems using GGA and HSE06 functionals.

Materials	Band gap (eV)		GGA		HSE06	
	GGA	HSE06	$m_h^*/m_0$	$m_e^*/m_0$	$m_h^*/m_0$	$m_e^*/m_0$
$\hbox {Ni}_{0.125}\hbox {Co}_{0.875}$S	1.628	1.840	1.149	0.366	1.200	0.380
$\hbox {Ni}_{0.250}\hbox {Co}_{0.750}$S	1.590	1.800	1.159	0.375	1.210	0.385
$\hbox {Ni}_{0.375}\hbox {Co}_{0.625}$S	1.550	1.750	1.252	0.382	1.260	0.390
$\hbox {Zn}_{0.125}\hbox {Co}_{0.875}$S	1.450	1.680	1.095	0.400	1.100	0.410
$\hbox {Zn}_{0.250}\hbox {Co}_{0.750}$S	1.400	1.630	1.220	0.403	1.230	0.415
$\hbox {Zn}_{0.375}\hbox {Co}_{0.625}$S	1.340	1.600	1.228	0.416	1.240	0.420
$\hbox {(Ni, Zn)}_{0.25}\hbox {Co}_{0.75}$S	1.214	1.610	1.056	0.455	1.060	0.460

### Electron cloud density

**Fig. 6:**
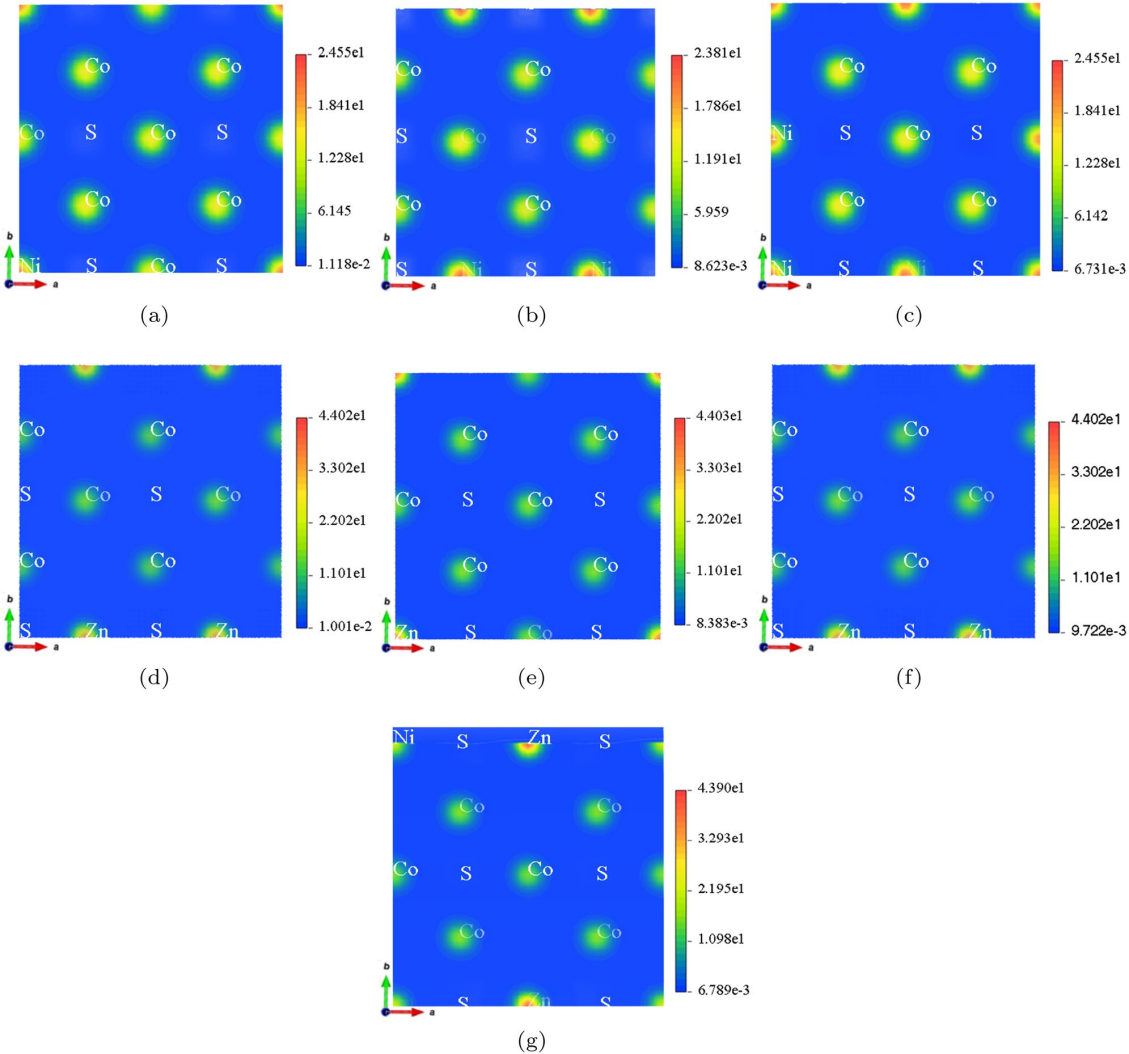
Top view of 2 $\times$ 2 $\times$ 1 supercell electron cloud density of (**a**) $\hbox {Ni}_{0.125}\hbox {Co}_{0.875}$S, (**b**) $\hbox {Ni}_{0.25}\hbox {Co}_{0.75}$S, (**c**) $\hbox {Ni}_{0.375}\hbox {Co}_{0.625}$S, (**d**) $\hbox {Zn}_{0.125}\hbox {Co}_{0.875}$S, (**e**) $\hbox {Zn}_{0.375}\hbox {Co}_{0.625}$S, (**f**) $\hbox {Zn}_{0.25}\hbox {Co}_{0.75}$S, (**g**) $\hbox {(Ni, Zn)}_{x}\hbox {Co}_{1-x}$S ($x = 0.125$). The color maps represent electron cloud density, with red regions indicating high electron density and blue regions indicating low electron density. The color bars on the right provide quantitative values for the electron density at each point.

The density of the electron cloud, or the charge density distribution, reflects the probability of finding electrons around atoms in a material^42–44^. Figure 6 presents the electron cloud density distributions for the Ni, Zn, and co-doped CoS crystal structures analyzed using Materials Studio, illustrating the effects of Ni and Zn on the charge distributions at various doping concentrations. In $\hbox {(Ni, Zn)}_{x}\hbox {Co}_{1-x}$S systems, a higher electron density (represented by red/yellow regions) corresponds to an increased charge localization, which implies a stronger bonding with a more covalent nature. Conversely, a lower electron density (indicated by the blue medium) signifies electron depletion and weaker interactions, indicating a more ionic character. As shown in Fig. 6 (a), the high electron density around Co and S arises due to the partial substitution of Co by Ni. On the other hand, the reduced electron density at the Ni sites suggests that the Ni–S bonds are weaker than the Co–S bonds. Figure 6 (b) illustrates that in $\hbox {Ni}_{0.25}\hbox {Co}_{0.75}$S, the incorporation of Ni alters the charge distribution, leading to a reduction in charge localization. This change can have a significant impact on the material’s conductivity. Figure 6 (c) shows that $\hbox {Ni}_{0.375}\hbox {Co}_{0.625}$S exhibits a more uniform charge distribution, which reduces localization and may enhance charge transport properties. Figure 6 (d) illustrates that Zn doping introduces localized variations in electron density around Co and Zn atoms within the material. The presence of Zn atoms results in a weaker electron cloud distribution than Ni, highlighting these elements’ distinct electronic contributions. These subtle changes in charge density indicate that Zn has a lower electron affinity than Ni, which significantly influences the overall electronic properties of the doped system. In Figure 6 (e), $\hbox {Zn}_{0.125}\hbox {Co}_{0.875}$S displays a lower electron density for Zn compared to Co, indicating weaker Zn-S interactions and greater charge delocalization, potentially improving electron mobility. Figure 6 (f) reveals that $\hbox {Zn}_{0.25}\hbox {Co}_{0.75}$S has more localized electron density around Co than around Zn, suggesting that the influence of Zn on charge redistribution is less significant than that of Ni atoms. Figure 6 (g) illustrates that in $\hbox {(Ni, Zn)}_{x}\hbox {Co}_{1-x}$S, the combined effects of Ni and Zn doping enhance charge delocalization, particularly around sulfur atoms. This synergistic effect leads to substantial modifications in the material’s electronic structure and electron transport properties.

### Optical properties

**Fig. 7:**
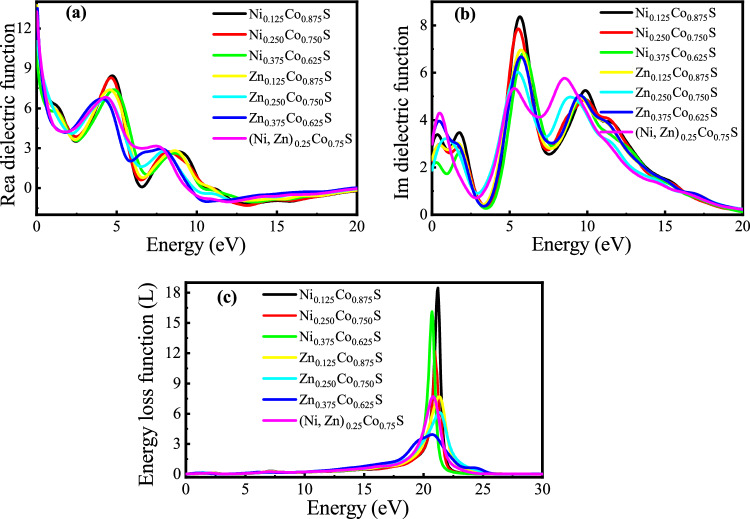
(**a**) Calculated real part of the dielectric function, (**b**) imaginary part of the dielectric function, (**c**) electron energy loss function spectra (EELs) of Ni, Zn, and co-doped CoS crystals.

In this subsection, the optical properties of the compounds were studied by examining several factors: dielectric function, refractive index, extinction coefficient, optical conductivity, absorption coefficient, and electron energy loss. The direct band gap of the studied compounds in the visible region enhances their potential for optoelectronic and solar cell applications^45^. The optical properties are described by the complex dielectric function $\varepsilon (\omega ) = \varepsilon _1(\omega ) + \varepsilon _2(\omega )$, where $\varepsilon _1(\omega$) and $\varepsilon _2(\omega )$ are the real and imaginary parts related to the band structure^46,47^. The DFT with a 300 k-mesh was used to calculate the dielectric and optical properties for all doping concentrations. Figure 7 shows the results for Ni, Zn, and co-doped CoS, specifically the real and imaginary components of the dielectric function and the energy loss function (ELF) within the examined energy range. This analysis offers valuable insights into the optical behavior of Ni, Zn, and co-doped CoS and their potential use in optoelectronic devices.

The data emphasize the impact of doping on the material’s optical behavior. The real part of the dielectric function reflects the material’s ability to polarize in response to an electric field, which affects light refraction. In Ni-doped CoS, distinct peaks are observed in the low energy range (below 5 eV), indicating the high static polarizability. Zn doping leads to a slightly elevated refractive index, roughly around 4 eV, which may influence the material’s optical transparency. Co-doped samples show a more balanced response, characterized by reduced peak intensities compared to those of individually doped samples, suggesting a synergistic interaction between the Ni and Zn dopants. The static dielectric constants for Ni-doped CoS were measured as 12.69, 12.25, and 11.79 at Ni concentrations of 0.125, 0.25, and 0.375, respectively. For Zn and co-doped samples, the static dielectric constants were 13.73, 12.97, 12.20, and 13.26 for respective codoping concentrations of 0.125, 0.25, 0.375, and 0.125.

The imaginary part of the dielectric function corresponds to the energy absorption caused by interband electronic transitions. For Ni-doped CoS, prominent peaks at approximately 5 eV and 10 eV indicate strong absorption attributed to transitions involving Ni-induced states in the conduction band. In contrast, Zn-doped CoS displays slightly lower peaks. The samples maintain notable absorption features near 5 eV while showing a reduced absorption intensity at higher energies. This decrease in intensity indicates an improved optical efficiency for specific applications. The calculated values of the static dielectric function, refractive index, and absorption coefficient for the $\hbox {(Ni, Zn)}_{x}\hbox {Co}_{1-x}$S (x=0.125) systems at zero photon energy are summarized in Table 4.

The energy loss function reflects energy dissipation caused by plasma oscillations, prominently reflecting the plasma resonance energy. All samples exhibit a sharp peak within the 20–22 eV range, corresponding to the plasma resonance frequency where energy loss is highest. Compared to single-doped samples, codoped samples show a slightly broader loss function, suggesting enhanced stability against energy dissipation. This property is essential for applications requiring minimal energy loss, such as optoelectronic devices.

**Table 4:** Calculated optical properties of $\hbox {(Ni, Zn)}_{x}$)$\hbox {Co}_{1-x}$S under different dopant concentrations at zero photon energy.

Crystal Composition	Static Dielectric Constant	Refractive Index	Absorption Coefficient
$\hbox {Ni}_{0.125}\hbox {Co}_{0.875}$S	12.69	3.87	2.91
$\hbox {Ni}_{0.250}\hbox {Co}_{0.750}$S	12.25	3.93	2.27
$\hbox {Ni}_{0.375}\hbox {Co}_{0.625}$S	11.79	3.97	2.17
$\hbox {Zn}_{0.125}\hbox {Co}_{0.875}$S	13.73	3.72	2.33
$\hbox {Zn}_{0.250}\hbox {Co}_{0.750}$S	12.97	4.80	2.14
$\hbox {Zn}_{0.375}\hbox {Co}_{0.625}$S	12.20	4.92	2.12
$\hbox {(N, Zn)}_{0.25}\hbox {Co}_{0.75}$S	13.26	4.05	2.07

#### Optical conductivity

**Fig. 8:**
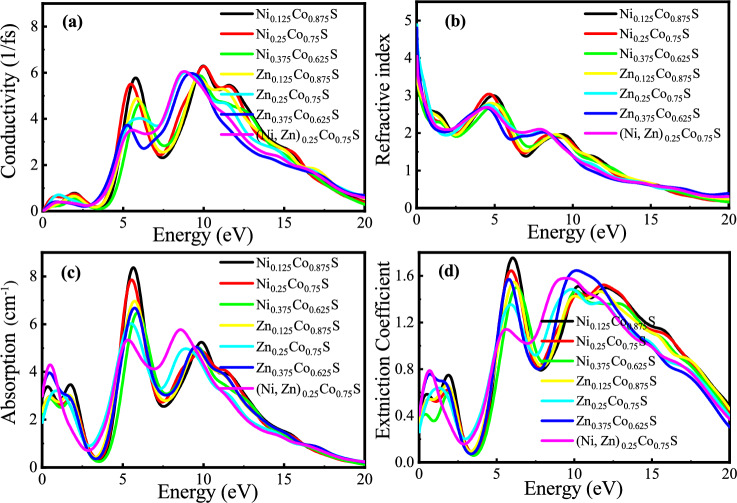
(**a**) conductivity, (**b**) refractive index, (**c**) absorption coefficient, and (**d**) extinction coefficient of Ni, Zn, and co-doped CoS crystal.

The optical conductivity of Ni, Zn, and co-doped CoS CE materials for DSSCs, crucial for effective counter electrode performance, is directly related to their band gap properties. While Ni doping leads to a higher band gap and enhanced UV-visible conductivity, promoting efficient charge transfer, Zn doping can have the opposite effect. Codoping allows tuning the band gap and, consequently, the optical conductivity to an intermediate level, optimizing charge transfer at the CE. This balance makes co-doped CoS promising for improving DSSC efficiency by facilitating efficient charge regeneration. The optical conductivity, $\sigma (\omega )$, derived from the imaginary part of the dielectric function, is given by^48^:

3 $$\begin{aligned} \sigma (\omega ) = \frac{\omega \epsilon _2(\omega )}{4\pi }, \end{aligned}$$

where ($\omega$) denotes the angular frequency of the material. Figure 8 presents the optical properties of Ni, Zn, and Ni-Zn co-doped CoS, specifically conductivity, refractive index, absorption, and extinction coefficient. The different doping concentrations significantly affect these properties across the energy range, especially at high-energy peaks. These properties are essential for assessing the materials’ potential as counter electrodes in DSSCs.

#### Absorption coefficients

Unlike photoanodes that rely on optical absorption to generate charge carriers, counter electrodes prioritize electrical conductivity, electrocatalytic activity, and stability rather than solar absorption. While comparing their absorption characteristics with the solar spectrum may indicate potential parasitic losses, this is not a primary performance metric. However, minimal visible-light absorption could help mitigate unwanted photovoltage losses or side reactions. The absorption coefficient, $\alpha$($\omega$) can be calculated using the following formula^49^:

4 $$\begin{aligned} \alpha (\omega ) = \frac{4\pi k}{\lambda }, \end{aligned}$$

where denotes $k$ is the extinction coefficient, and $\lambda$ is the wavelength of the incident light. Examining the absorption coefficient offers insight into the electronic structure and, consequently, the optical properties of the material.

This study investigated the absorption coefficients of Ni, Zn, and co-doped CoS at various doping concentrations. The results revealed that Ni-doped CoS had a higher absorption coefficient due to its wider band gap, which enhanced light harvesting in the UV-visible range and made it particularly effective as a counter electrode in DSSCs. Conversely, Zn doping led to a smaller band gap and a reduced absorption coefficient, resulting in weaker light interaction, especially in the UV region, which diminishes the effectiveness of Zn-doped materials in light-harvesting applications compared to Ni-doped materials. Co-doped CoS achieved an optimal balance by combining the advantages of both Ni and Zn, providing an intermediate absorption coefficient that enhances overall light absorption and energy conversion efficiency. This tunability in absorption properties positions co-doped CoS as a promising candidate for improved photovoltaic performance in DSSCs.

#### Refractive index

The refractive index, $n(\omega )$, a fundamental parameter describing a material’s optical properties, quantifies how much light slows down due to light-electron interactions. It is related to the optical properties of the material, particularly refraction and absorption, via the complex dielectric function, which consists of real, $\varepsilon _1(\omega )$ and imaginary, $\varepsilon _2(\omega )$ parts. The refractive index was determined using the following equation^50^.

5 $$\begin{aligned} n(\omega ) = \frac{1}{\sqrt{2}}\left( \sqrt{\epsilon ^{2}_{1}(\omega ) + \epsilon ^{2}_{2}(\omega )} + \epsilon _{1}(\omega )\right) ^{\frac{1}{2}} \end{aligned}$$

For Ni, Zn, and co-doped CoS, the refractive index reflects their optical behavior. The higher band gap in Ni-doped CoS leads to a reduced refractive index, influencing device performance in ways that may not directly involve enhancing light confinement. Conversely, Zn-doped CoS, having a smaller band gap, has a higher refractive index, potentially affecting light interaction. Co-doping offers a balanced approach, resulting in an intermediate refractive index and enabling optimized light absorption and improved device efficiency. This tunability positions co-doped CoS as a promising material for advanced photovoltaic technologies.

#### Extinction coefficient

The extinction coefficient is a fundamental parameter for assessing the optical properties of materials, particularly their ability to absorb light. It is a key indicator of how effectively a material interacts with light and can be determined by the following equation^51^:

6 $$\begin{aligned} k(\omega ) = \frac{1}{\sqrt{2}}\left( \sqrt{\epsilon ^{2}_{1}(\omega ) + \epsilon ^{2}_{2}(\omega )} - \epsilon _{1}(\omega )\right) ^{\frac{1}{2}} \end{aligned}$$

where $\epsilon _{1}(\omega )$ and $\epsilon _{2}(\omega )$ denote the real and imaginary components of the dielectric function, respectively. This equation underscores the significance of the dielectric function in analyzing and quantifying the absorption behavior of materials. The extinction coefficient, which quantifies a material’s ability to absorb light, is closely linked to its band gap. Typically, materials with higher band gaps exhibit higher extinction coefficients in the high-energy (UV-visible) range, as they are more efficient at absorbing photons of those energies. Ni-doped CoS, characterized by the higher band gap, demonstrates the highest extinction coefficient, indicating strong absorption in the UV-visible region. In contrast, Zn-doped CoS, with the smallest band gap, shows a lower extinction coefficient, particularly in the UV range, suggesting weaker light absorption capabilities. The co-doped CoS materials, which possess an intermediate band gap between the Ni-doped and Zn-doped samples, also exhibit a moderate extinction coefficient. This balanced characteristic of co-doped CoS enables the tuning of absorption properties, thereby optimizing light interaction and enhancing the overall performance of DSSCs.

#### Reflectivity and transmittance

**Fig. 9:**
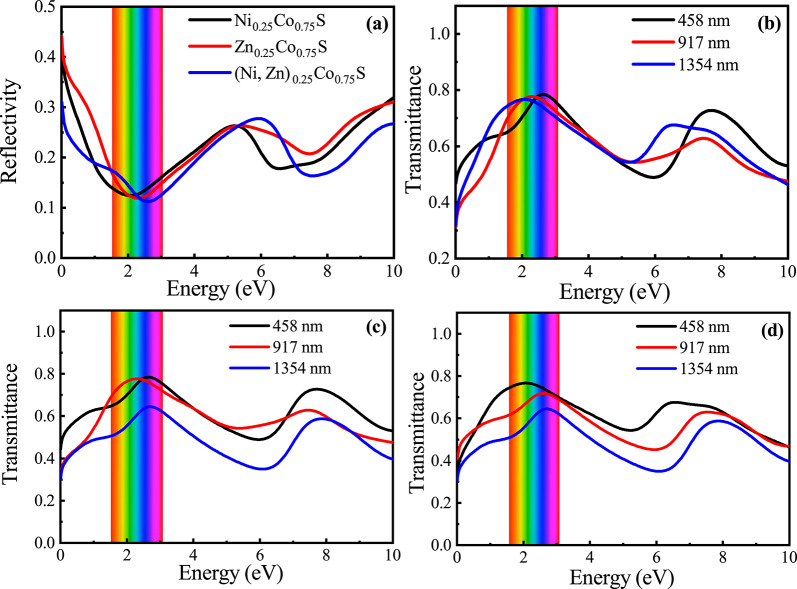
(**a**) Reflectivity across the visible to near-infrared photon energy range for $\hbox {(Ni, Zn)}_{x}\hbox {Co}_{1-x}$S, (**b**–**d**) transmittance as a function of photon energy for $\hbox {Ni}_{0.25}\hbox {Co}_{0.75}$S, $\hbox {Zn}_{0.25}\hbox {Co}_{0.75}$S, and $\hbox {(Ni, Zn)}_{0.25}\hbox {Co}_{0.75}$S at different sample thicknesses.

Using the dielectric function, the reflectivity (*R*) and transmittance (*T*) of the alloy are determined as a function of photon energy and presented in Fig. 9 (a-d). The transmittance *T*(*d*) of the $\hbox {(Ni, Zn)}_{x}\hbox {Co}_{1-x}$S alloy, depending on both the sample thickness (*d*) and photon energy, is calculated according to the formula^21,22^,

7 $$\begin{aligned} T(d) = (1 - R) e^{-\alpha d}, \end{aligned}$$

where $R$ is the reflectivity, $\alpha$ is the absorption coefficient, and $d$ is the film thickness. For the most transparent sulfide thin-film devices, thicknesses are adopted from reported experimental studies in the literature^16^. Figure 9 (a) reveals that all three materials exhibit very low reflectivity consistently below 0.5 % over the entire energy spectrum, indicating minimal light reflection and strong potential for high optical transmission. Figure 9 (b-d) depicts the transmittance behavior at three specific energies corresponding to wavelengths of 458 nm, 917 nm, and 1354 nm for each material, respectively. All samples demonstrate high transmittance, typically exceeding 60 %, with the highest values observed at lower energies.

### Prediction of photovoltaic performance

The computational analysis assesses the photovoltaic performance of Ni, Zn, and co-doped CoS as counter electrode materials. Among the single and co-doped systems, co-doped CoS exhibits superior performance, outperforming Ni-doped CoS, which in turn performs better than Ni and Zn-doped CoS systems. This trend is governed by critical device parameters, particularly the fill factor (FF), short-circuit current density ($\hbox {J}_{sc}$), and overall power conversion efficiency (PCE). The improved performance of Ni-Zn co-doped CoS is attributed to enhanced electronic and optical properties that facilitate charge transfer kinetics while reducing parasitic losses. A key factor behind the enhanced performance of co-doped CoS is the improvement in both electronic and optical properties, which collectively facilitate faster charge transfer kinetics and minimize parasitic losses. The fill factor, in particular, is significantly improved due to the reduced charge transfer resistance ($R_{ct}$) at the CE/electrolyte interface, expressed as follows^52^:

8 $$\begin{aligned} FF = \frac{J_{max}\times V_{max}}{J_{sc}V_{oc}}, \end{aligned}$$

where $V_{max}$ and $J_{max}$ represent the voltage and the current per unit area at the maximum output power point, respectively. The co-doped material demonstrates enhanced electrical conductivity and charge transfer due to band gap tuning, resulting in lower $R_{ct}$ and $R_{s}$, significantly boosting the FF.

Furthermore, $\hbox {J}_{sc}$ benefits indirectly from the co-doping strategy through the moderate absorption coefficient and extinction coefficient of co-doped CoS, which minimizes unwanted light absorption at the CE and allows more electrons to reach the dye-sensitized photoanode. The overall power conversion efficiency is synergistically improved through combining in both $J_{sc}$ and FF, as expressed by:

9 $$\begin{aligned} \eta = \frac{J_{sc} \cdot V_{oc} \cdot FF}{P_{in}} \times 100\%, \end{aligned}$$

where $P_{in}$ denotes the incident light power. While $V_{oc}$ may be less directly influenced, the reduction in recombination and improved interfacial charge collection in co-doped systems help maintain or slightly elevate $V_{oc}$. Thus, the balanced optical and electronic properties of Ni-Zn co-doped CoS enable optimal device performance, positioning it as the most effective counter electrode material in this study, with the highest predicted PCE due to its ability to maximize $J_{sc}$ and FF without compromising stability or transparency.

10 $$\begin{aligned} \text {PCE} = \frac{V_{oc} \times J_{sc} \times FF}{P_{in}}, \end{aligned}$$

where $V_{oc}$ is the open-circuit voltage and $P_{in}$ is the incident solar power. While $V_{oc}$ is less directly affected by the CE’s optical properties, the reduced recombination and improved charge regeneration in co-doped systems can contribute to its stability or slight enhancement.

## Conclusion

The first-principles investigation based on density functional theory (DFT), employing both the generalized gradient approximation (GGA) and the hybrid HSE06 functional within the Quantum ESPRESSO framework, has been conducted to elucidate the structural, electronic, and optical properties of $\hbox {(Ni, Zn)}_{x}\hbox {Co}_{1-x}$S systems. The optimized lattice parameters and bond lengths of single and co-doped show that Ni doping shortens the Ni–S bond, enhancing electron localization, while Zn doping weakens the bond and promotes electron delocalization. Moreover, defect formation energy calculations yield negative values, which are indicative of the thermodynamic stability of the systems. The analysis electronic band structure reveals that all doped systems display a direct band gap, with a progressive decrease in the band gap upon doping. Notably, a continuous reduction is observed from single doping to co-doping, culminating in the lowest band gap value for the co-doped system. This observation indicates successful band gap engineering through alloying. The trend is consistent across both the GGA and HSE06 hybrid functional calculations, confirming the enhanced electronic tunability achieved through the incorporation of Ni and Zn. These finding is confirmed with the calculation of the effective mass. The valence band is primarily composed of Co(3d), S(3p), and Ni(3d) orbitals, and a high contribution of Zn(4s) orbitals enhances hybridization near the valence band edge. Enhanced dielectric constants in doped CoS improve charge carrier mobility and light harvesting capabilities, as evidenced by absorption and conductivity spectra showing strong UV-visible range performance and good electrical conductivity. Furthermore, upon the assessment of the band gap tuning and optical analysis $\hbox {(Ni, Zn)}_{x}\hbox {Co}_{1-x}$S materials, the photovoltaic performance was predicted. These results emphasize the co-doped material’s adjustable electronic and optical characteristics, positioning it as a promising option for applications like CEs in DSSCs. Based on our DFT results, we recommend that experimental validation using the same single, and co-doping concentrations is essential for real-world DSSCs applications. 

## Data Availability

All data provided are included in the manuscript.
